# Mitochondrial DNA variations and mitochondrial dysfunction in Fanconi anemia

**DOI:** 10.1371/journal.pone.0227603

**Published:** 2020-01-15

**Authors:** Avani Solanki, Aruna Rajendran, Sheila Mohan, Revathy Raj, Babu Rao Vundinti

**Affiliations:** 1 Department of Cytogenetics, National Institute of Immunohaematology, K.E.M. Hospital Campus, Parel, Mumbai, Maharashtra, India; 2 Department of Hematology, Institute of Child Health and Hospital for Children, Egmore, Chennai, Tamil Nadu, India; 3 Pediatric Haematology Department, Apollo Children’s Hospital, Chennai, Tamil Nadu, India; Yale University, UNITED STATES

## Abstract

*In-vitro* studies with different Fanconi anemia (FA) cell lines and *FANC* gene silenced cell lines indicating involvement of mitochondria function in pathogenesis of FA have been reported. However, *in-vivo* studies have not been studied so far to understand the role of mitochondrial markers in pathogenesis of FA. We have carried out a systematic set of biomarker studies for elucidating involvement of mitochondrial dysfunction in disease pathogenesis for Indian FA patients. We report changes in the mtDNA number in 59% of FA patients studied, a high frequency of mtDNA variations (37.5% of non-synonymous variations and 62.5% synonymous variations) and downregulation of mtDNA complex-I and complex-III encoding genes of OXPHOS (p<0.05) as strong biomarkers for impairment of mitochondrial functions in FA. Deregulation of expression of mitophagy genes (ATG; p>0.05, Beclin-1; p>0.05, and MAP1-LC3, p<0.05) has also been observed, suggesting inability of FA cells to clear off impaired mitochondria. We hypothesize that accumulation of such impaired mitochondria in FA cells therefore may be the principal cause for bone marrow failure (BMF) and a plausible effect of inefficient clearance of impaired mitochondria in FA.

## Introduction

Mitochondria are powerhouse of cells and generate energy in the form of ATP through oxidative phosphorylation process. Mitochondria are also source of reactive oxygen species (ROS) production. Thus mitochondrial dysfunction is detrimental to the organism [1]. Previous studies have established link between mitochondrial functions and oxygen metabolism in FA. There is also an evidence of involvement of FA proteins in mitochondrial dysfunctions. Mukhopadhyay et al., 2006 found that the FANCG protein is localized in mitochondria and interacts with the mitochondrial peroxidase peroxiredoxin3 (PRDX3). In turn, cells from FA-A and FA-C subtypes also had PRDX3 cleavage and decreased peroxidase activity. These findings further supported the idea of mitochondrial involvement in the pathogenesis of FA [2]. Kumari et al., 2013 have demonstrated decrease of mitochondrial membrane potential, low ATP production, impaired oxygen consumption rate and pathological changes in the morphology of FA mitochondria. The study also showed inactivation of the enzymes responsible for energy production, detoxification of ROS and over sensitivity to DNA cross-linkers by the overproduction of ROS [3,4].

Ravera et al., 2013 have analysed the respiratory fluxes in FANCA primary fibroblasts, lymphocytes and lymphoblasts. Their study revealed that *FANCA* mutants show defective respiration through Complex I, diminished ATP production and metabolic sufferance with an increased AMP/ATP ratio. Treatment with N-acetyl cysteine (NAC) restored oxygen consumption to normal level [5]. Recently it was also shown that genetic deletion of Fancc blocks the autophagic clearance of viruses (virophagy) and increases susceptibility to lethal viral encephalitis. FANCC protein interacts with Parkin, is required in vitro and in vivo for clearance of damaged mitochondria, and decreases mitochondrial ROS production and inflammasome activation, ultimately leading to phenotypes such as BMF, cancers, and aging associated with mutations in FA pathway genes [6,7].

Despite of experimental evidences on FA cell lines or *FANC* gene silenced cells for accumulation of impaired mitochondrial activities and deregulated mitophagy, there has been no biomarker study done to elucidate involvement of mitochondrial dysfunction in FA pathogenesis. Here, we present study of mitochondrial dysfunction and impaired mitophagy in *ex-vivo* studies on mononuclear cells derived from peripheral blood of FA patients and also mitochondrial DNA (mtDNA) variations to understand the genetic basis of mitochondrial DNA pathogenesis in FA.

## Patients and materials and methods

### Patients, specimen collection and ethical clearance

*Seventy* FA subjects including 42 males and 28 females with a mean age of 8 years were included in the study (March, 2012 to June, 2017). The peripheral blood samples were collected in heparin (7cc) and EDTA (4cc) vacutainers from the patients and 33 age-matched healthy controls (non-FA or individuals without history of haematological abnormalities that are found in FA) with the written consent of parents (in case of minors) and adult patients and controls. The study protocols were approved by Institutional Ethics Committee for human subjects of National Institute of Immunohaematology, Parel, Mumbai. The diagnosis for FA was confirmed with the clinical examination and methods described in our previous publications for investigations like chromosomal breakage [8], FANCD2 immunoblotting [8] and complementation group determination using direct sequencing [8] and targeted exome sequencing (TES). Molecular investigation for *FANC* genes for a few patients was carried at MedGenome Pvt. Ltd., Karnataka, India and with collaboration with Dr. Minoru Takata’s Lab at Radiation Biology Center, Kyoto University, Kyoto, Japan. It was done by targeted gene capture using a custom capture kit. The libraries were sequenced on Illumina sequencing platform (mean coverage >80 to 100X). The identified mutations were confirmed by direct sequencing.

### qPCR based mtDNA copy number change

Genomic DNA (gDNA) was used for mtDNA copy number study. The genomic DNA was extracted from peripheral blood collected in EDTA vacutainers using QIAamp DNA Blood Midi Kit (Qiagen, cat.# 51183) according to the manufacturer's instructions. TaqMan Universal PCR mastermix (Thermo Fisher Scientific cat.# 4304437) was used with final concentration of 200nM probe and 900nM of forward and reverse primers. A series of 10 fold dilution of standard DNA was prepared for standard curve and 15ng of gDNA was added in sample wells. An initial 10 minutes denaturation at 95°C followed by 40 cycles of 95°C and combined annealing-extension at 60°C was standardized as run program on ABI StepOne machine. Formula used for copy number change = **2**^**– (mtDNA CT–gDNA CT)**^. A set of primers and probes was used to study mtDNA copy number change, and β2-microglobulin primers and probes for nuclear genome as described by Malik Sahani et al., 2011 [9].

### OXPHOS enzymes and *TFAM* expression using qPCR

RNA was extracted from EDTA peripheral blood using QIAamp RNA Blood Mini Kit (Qiagen, cat.#52304) according to the manufacturer's instructions. The concentrations of RNA were determined on nanodrop spectrophotometer. 1μg of RNA was reverse transcribed to first-strand cDNA by using RevertAid H minus First Strand cDNA Synthesis Kit (Thermo Scientific, cat.#K1632). For OXPHOS enzyme expression profiling was done by Real time PCR primers designed so as to amplify transcripts encoding OXPHOS enzymes (Complex I: NADH-coenzyme Q reductase, Complex III: CoenzymeQ—Cytochrome c reductase and *TFAM* were used (S1 Table). Kapa SYBR FAST qPCR Master Mix (2X) Green (cat# KM4103) was used to quantify transcripts on ABI StepOne machine.

### Study of mtDNA variations of OXPHOS Complex-I subunits and Complex-III encoding genes

Genomic DNA was extracted from peripheral blood stored in EDTA vacutainer using QIAamp blood midi kit (Qiagen, cat.# 51183) and was used for study of mtDNA variations. Primers for amplifying Complex-I subunits and Complex-III encoding genes were designed as given by Rieder et al., 1998 [10] and processed for direct sequencing to screen the variations as described in [8]. Mitomap database was used to compare the obtained sequence data (https://www.mitomap.org/).

### Mitophagy associated gene expression using qPCR

Relative quantification of mitophagy associated genes (*ATG12*, *BECLIN1*, and *MAP1-LC3*) was carried out with standard melt-curve protocol. The transcripts used were same as described in *OXPHOS enzyme expression using qPCR* section of methodology. The primers for *ATG12*, *BECLIN1*, and *MAP1-LC3* were used at 250nM, 300nM and 300nM respectively, with Kapa SYBR FAST qPCR Master Mix Green (Kapa Biosystems, cat.# KM4103) to quantify transcripts on ABI StepOne machine in 20μl reaction mixture. Primer sequences were designed as described by Cotan et al., 2011 [11].

### Statistical analysis

Statistical analysis was carried out using Graph Pad InStat 2 software (Graph Pad Software Inc., La Jolla, CA, U.S.A.). Statistical significance of different experiments performed for the study was determined either using Student’s t-test or using Chi-square test. A p-value ≤0.05 was considered statistically significant.

## Results

### mtDNA copy number change

**Fig 1:**
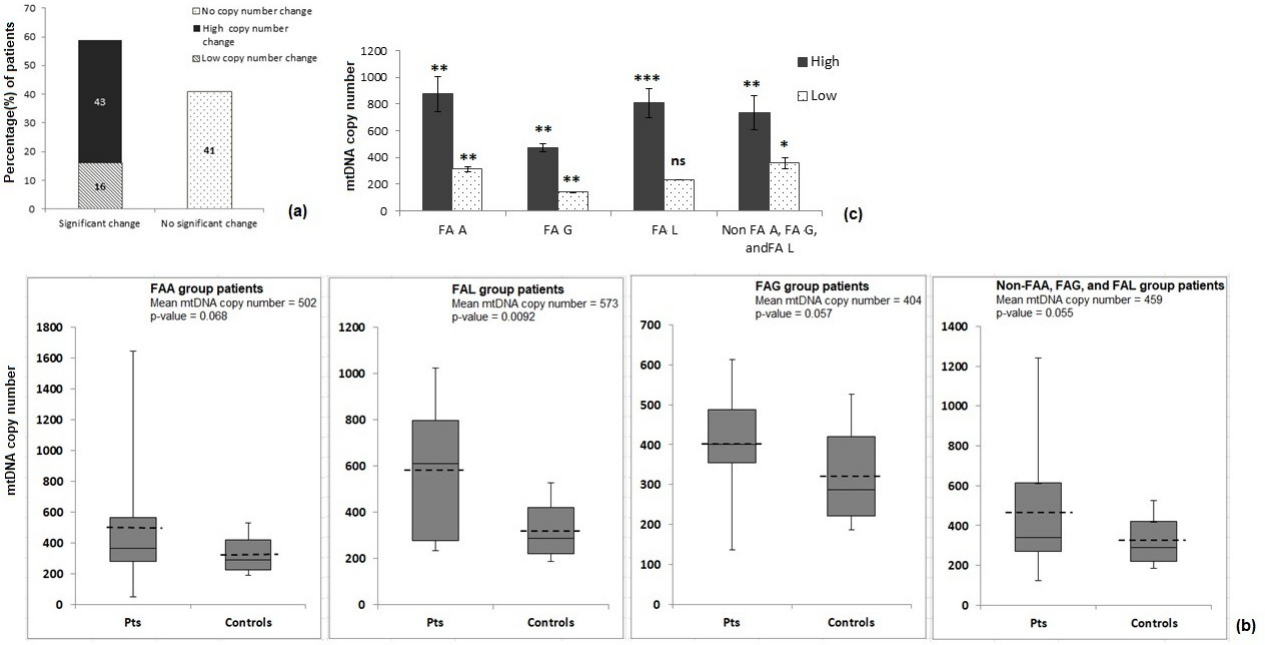
mtDNA copy number change study. (a) Frequency distribution of mtDNA copy number change among FA patients (%), (b) Whisker and box plot for mtDNA copy number change among different FA complementation group patients. Dashed line represents mean mtDNA copy number (Pts.: Patients), and (c) mtDNA copy number change (Low and High) among patients of different FA complementation groups (ns: not significant, *: p<0.05, **: p< 0.01, and ***: p<0.00001).

Patients with positive chromosomal breakage investigation were screened using FANCD2 immunoblot to locate upstream or downstream complex defect (S2–S7 Tables). Based on defect in the pathway, complementation groups were assigned to the patients by molecular investigations such as direct sequencing and TES (S2–S6 Tables). Of the 70 FA patients studied for mitochondrial DNA (mtDNA) copy number change, 29(41%) patients showed no significant change in mtDNA copy number compared to controls and 41(59%) patients showed significant change in mtDNA copy number– 11 (16%) patients with low mtDNA copy number or decrease in mtDNA copy number and 30 (43%) with high copy number or increase in copy number (Fig 1A). The mean copy number changes of mtDNA were found to be 502 (p = 0.068), 404 (p = 0.057) and 573 (0.0092) for FA-A, FA-G and FA-L group patients respectively (Table 1, Fig 1B). The mean values of high and low copy number changes among patients of different complementation groups were found to be statistically significant (p<0.05) (Table 2, Fig 1C).

**Table 1:** mtDNA copy number of FA patients from different complementation groups.

Complementation group	Mean	Std Dev	Std Err	t-value	p-value
FA-A (n = 36)	501.9504	426	74	1.51833	0.068
FA-G (n = 12)	404.3956	130	39	1.64734	0.057
FA-L (n = 8)	573.3908	331	125	2.58287	0.0092
Non-FA-A, -G, and -L (n = 14)	443.52	297	68	1.64378	0.055
Controls (n = 33)	323	115	31		

**Table 2:** mtDNA copy number change (Low and High) among patients of different FA complementation groups.

	mtDNA copy number	Mean	StdDev	Std. Err	t-value	p-value
FA-A	High (n = 13)	874.02	466.06	129.26	4.2894	0.000117
	Low (n = 5)	311	44.64	16.87	-3.9697	0.00041
FA-G	High (n = 7)	475.54	80.27	30.34	3.11507	0.00285
	Low (n = 1)	136	0	0	-2.2287	0.02136
FA-L	High (n = 4)	806.67	222.08	111.04	6.01901	<0.00001
	Low (n = 2)	233	0	0	-1.0748	0.15033
Non-FA-A, FA-G, and FA-L	High (n = 6)	736.08	314.19	128.27	4.39719	0.000174
	Low (n = 3)	354	59.58	42.13	-1.7897	0.04758

### Study of mtDNA variations for OXPHOS complex-I and complex-III encoding genes

**Fig 2:**
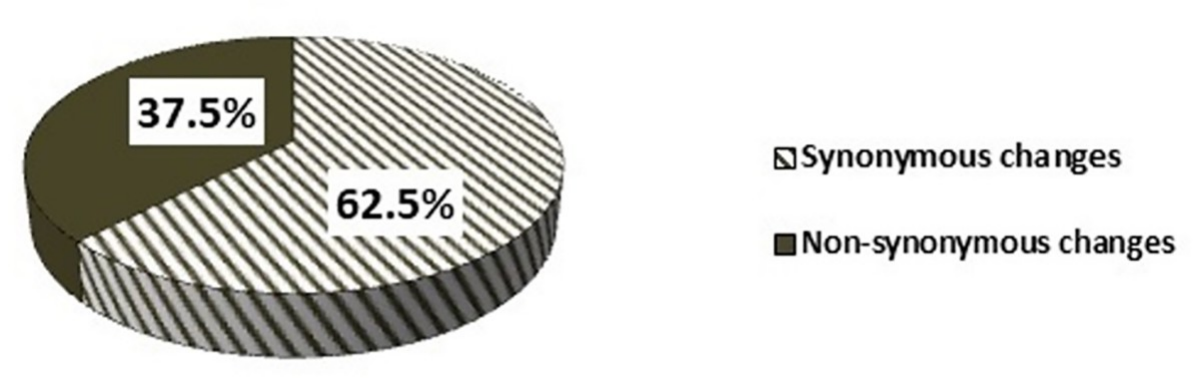
Types and frequency of mtDNA variations detected in Complex-I subunits and Complex-III encoding genes of OXPHOS.

A total of 184 (115 synonymous and 69 non-synonymous) mtDNA variations of complex I subunits and complex III encoding genes of OXPHOS were detected in our study (Fig 2). Of 184 mtDNA variations, 138 (76%) were different variants and only 46 (24%) of variations were found to be frequently occurring among the FA patients. Majority of variations were results of transition changes (177/184, 96%) in the bases of DNA and a very few were transversion changes (7/184, 4%) (Table 3). Some of the frequently occurring synonymous and non-synonymous mtDNA variations observed in the study were screened to analyse if their occurrence is statistically significant compared to controls (Tables 4 and 5).

**Table 3:** Frequency (%) of transitions and transversions observed in different types of mtDNA variations.

	Synonymous changes (n = 115)	Non-synonymous changes (n = 69)
Transitions	112 (97.54%)	65 (94.20%)
Transversions	3 (2.46%)	4 (5.80%)

**Table 4:** mtDNA non-synonymous variations of OXPHOS complex-I subunits and complex-III encoding genes.

Non-synonymous changes	GENES	AMINO ACID CHANGE	Transition/ Transversion	Frequency (%)	*X*^2^ score	p-value
4216 T>C	ND1	Y-H	Transition	14	4.7107	0.029976
4225 A>G	ND1	M-V	Transition	3	4.0313	0.044664
4917 A>G	ND2	N-D	Transition	2	5.6738	0.017221
5319 A>G	ND2	T-S	Transition	2	2.1978	0.138208
10398 A>G	ND3	T-A	Transition	44	4.5816	0.032317
14323 G>A	ND6	N-I	Transition	2	60	<0.05

**Table 5:** mtDNA synonymous variations of OXPHOS complex-I subunits and complex-III encoding genes.

Synonymous changes	Gene	Amino acid change	Transition/ Transversion	Frequency (%)	*X*^2^ score	p-value
4769 A>G	ND2	M-M	Transition	9	131.2331	<0.05
10400 C>T	ND3	T-T	Transition	43	15.7051	0.000074
10873 T>C	ND4	P-P	Transition	25	52.0252	<0.05
11083 A>G	ND4	M-M	Transition	2	52.0252	<0.05
11467 A>G	ND4	L-L	Transition	5	1.8018	0.179495
11812 A>G	ND4	L-L	Transition	2	5.6738	0.017221
12007 G>A	ND5	W-W	Transition	7	0.5786	0.446865
12372 G>A	ND5	L-L	Transition	2	5.6738	0.017221
12705 C>T	ND5	I-I	Transition	7	108.4122	<0.05
14905 G>A	CYTB	M-M	Transition	2	5.6738	0.017221
15043 G>A	CYTB	G-G	Transition	16	82.0513	<0.05
15301 G>A	CYTB	L-L	Transition	32	46.7532	<0.05
11251 A>G	CYTB	L-L	Transition	2	5.6738	0.017221

### Mitophagy associated gene expression profiling

**Fig 3:**
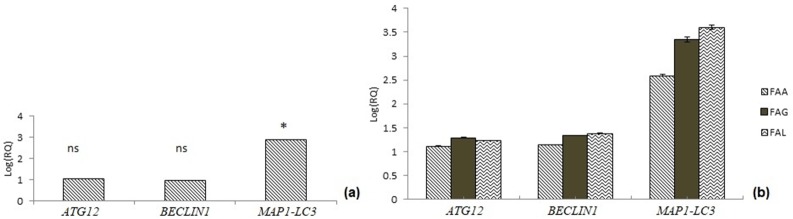
Study of mitophagy gene expression profiling. (a) Gene expression for *ATG12*, *Beclin1* and *MAP1-LC3* genes (Refer S8 Table for p-values), (b) Expression fold change for mitophagy genes (*ATG12*, *BECLIN1* and *MAP1-LC3*) among FA patients of different complementation group (Refer S9 Table for p-values).

A significant (p = 0.02019) fold change (2.9 fold change) was observed in *MAP1-LC3* gene expression of FA patients compared to controls. However, there was no significant difference in gene expression fold change (p>0.05) of *Beclin1* and *ATG12* genes of FA patients compared to controls (S8 Table and Fig 3A). Expression of *ATG12* and *Beclin1* genes among different FA complementation groups showed no significant (p>0.05) expression fold change (Fig 3B and S8 Table). Analysis of mitophagy expression according to distribution of patients among different complementation groups revealed a significant expression fold changes for *MAP1-LC3* gene in FA-L group patients (3.6 fold change, p<0.05) compared to FA-A (~2.6 fold change, p = 0.082) and FA-G group patients (3.3 fold change, p = 0.074) (Fig 3B and S9 Table).

### OXPHOS enzyme encoding genes and TFAM gene expression

**Fig 4:**
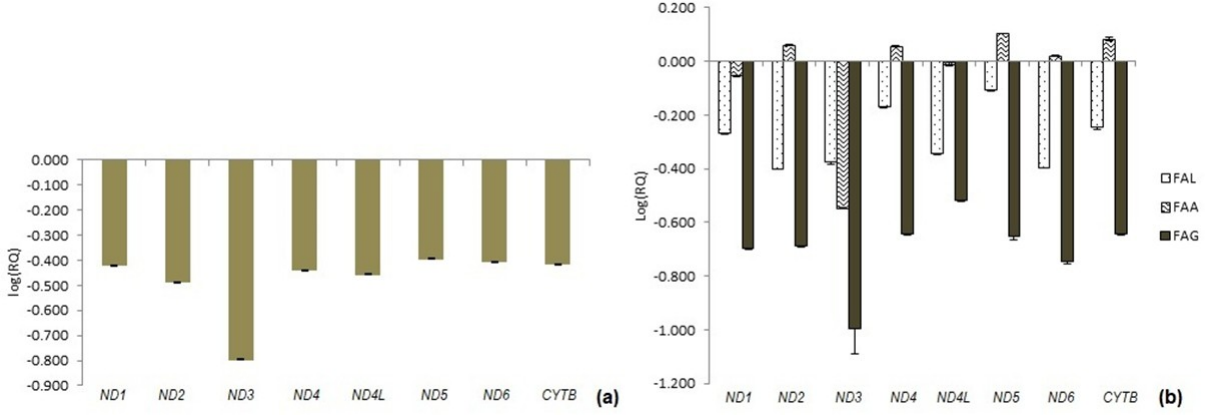
Expression profiling study for OXPHOS complex-I subunits and complex-III encoding genes. (a) Gene expression profiling for OXPHOS complex-I subunits and complex-III encoding genes in FA patients (Refer S10 Table for p-values), (b) OXPHOS complex-I subunits and complex-III encoding gene expression in FA patients of different complementation groups (Refer S11 Table for p-values).

Fig 4 and S10 Table are showing the expression of OXPHOS complex-I subunits (*ND1*, *ND2*, *ND3*, *ND4*, *ND4L*, *ND5*, and *ND6*) and Complex-III (*CYTB*) encoding genes. The study revealed significant downregulation in expression compared to controls (*ND1*, p = 0.0132; *ND2*, p = 0.0072; *ND3*, p = 0.02268; *ND4*, p = 0.01765; *ND4L*, p = 0.00914; *ND5*, p = 0.04515; *ND6*, p = 0.02335; *CYTB*, p = 0.01538) (Fig 4A and S10 Table). However, no specific trend was observed in expression change of genes encoding OXPHOS complex-I subunits and complex-III encoding genes of FA patients among different complementation groups (Fig 4B and S11 Table). Expression study for *TFAM* gene in FA patients showed ~7.66 fold increase in levels compared to controls (S1 Fig). Complementation group-wise cross-sectional analysis showed significant upregulation in expression of *TFAM* gene in FA-A, FA-G and FA-L complementation groups (S1 Fig).

## Discussion and conclusions

FA is a rare genetic disorder and presented with spectrum of clinical features [12]. Molecular studies have identified 22 genes associated with FA phenotype [13]. The bone marrow failure is one of the major clinical presentations in FA. However there have been several experimental proofs with no consensus result for one molecular mechanism cause underlying the BMF. Mitochondrial DNA (mtDNA) is known to be constantly challenged with ROS generated during electron transport reactions in oxidative phosphorylation event. Additionally, mtDNA lack histones and DNA damage repair system for mtDNA are not as much evolved as nuclear DNA. mtDNA copy number change has been known to be affected in many mitochondrial dysfunction syndromes and cancer conditions [14–17]. In our study, the mtDNA copy number change was observed in 59% of the FA patients where majority of them showed high copy number changes than low copy number change which suggests mitochondrial dysfunction in FA. We also carried out expression study for *TFAM* gene (Transcription factor A for mitochondria), a key regulator that drives mitochondrial DNA replication and transcription), to understand mitochondrial biogenesis controlling factor has any role to play [18]. We found that expression of *TFAM* gene was significantly upregulated in FA patients (S1 Fig). This implies that high copy number changes could be the plausible effect of compensatory mechanism of cells to cope up with energy requirement [9]. Correlation of mtDNA copy number changes with FA complementation groups suggests more significant change in copy number in FA-L group patients than FA-A and FA-G group patients. Further evaluation of same sample size of each complementation groups can lead us to understand relevance of variations in the significance of mtDNA copy number change among FA patients from different complementation group.

Mitochondrial dysfunction syndrome studies have been carried out using study of mtDNA variations especially OXPHOS reaction complex-I (NADH dehydrogenase) and complex-III (Cytochrome b) which pump electrons across inner mitochondrial membrane and generate proton gradient, the potential gradient generated is then used by ATPase-6 complex to generate energy in the form of ATP. mtDNA variations of genes encoding OXPHOS complexes have been studied and reported to form molecular pathology underlying the mitochondrial dysfunction [19, 20]. In our study, 62.5% of synonymous and 37.5% of non-synonymous changes have been observed in Complex-I subunits (ND1-ND6) and complex-III (CYTB) encoding genes. These are studied for molecular changes in FA patients and majority of the variations were result of transition changes in the nucleoside triphosphates (purine to purine or pyrimidine to pyrimidine). mtDNA variations T4216C and 10398A allele in FA patients suggest their association with mitochondrial dysfunction, as observed in other pathological diseases [21, 22]. Various mitochondrial dysfunction diseases have been shown to have impaired regulation in expression of mitochondrial Complex-I and Complex-III of OXPHOS genes [23, 24]. Gene expression study of OXPHOS complex-I and complex-III by real-time PCR suggests significant downregulation of complex-I subunits (*ND1* to *ND6*) encoding genes and complex-III encoding genes. Various biochemical studies have been carried out for mitochondrial OXPHOS complex-I activity and have shown that the activity of complex-I is hampered in FA-A group patients [5]. Thus mtDNA copy number changes together with variations detected in mtDNA and qPCR study OXPHOS complex-I subunits and complex-III encoding genes should be considered as mitochondrial dysfunction biomarkers to track deterioration of mitochondrial associated phenotypes in FA patients.

Mitophagy is specific clearance of impaired mitochondria when cells are facing crisis. Cells prefer to clear off impaired mitochondria rather than being submissive to the cellular crisis and undergoing apoptotic cell deaths. Inefficient clearance of mitophagy events and accumulation of impaired mitochondria have been studied in FA cell lines [25].We have observed no significant changes in the expression of *ATG12* and *BECLIN1* genes. However, expression of *MAP1-LC3* gene was found to be upregulated by ~3-fold in FA patients compared to age matched controls. The upregulated MAP1-LC3 (a useful autophagosomal marker) indicates initiation of autophagy of impaired mitochondria but inadequate expression of ATG12 protein (produces vesicle extension and completion in phagophore formation during mitophagy) and BECLIN1 (plays a significant role in cellular homeostasis and cross-regulation between apoptosis and autophagy) suggest incomplete clearance of dysfunctional mitochondria [26]. This could be a reason for accumulation of impaired mitochondria in FA cells. No specific trend was observed in expression of *ATG12*, *Beclin1* and *MAP1-LC3* genes in patients from different complementation group, suggesting inefficient mitophagy to be a generalized event rather than be associated with specific FA complementation group.

Changes in the mtDNA number (in 59% of FA patients), a high frequency of mtDNA variations (37.5% of non-synonymous variations and 62.5% synonymous variations) and downregulation of mtDNA complex-I and complex-III encoding genes of OXPHOS (p<0.05) are strong biomarkers for impairment of mitochondrial functions in FA. Deregulation of mitophagy genes (*MAP1-LC3*, p<0.05) suggests inability of FA cells to clear off impaired mitochondria. Accumulation of such impaired mitochondria in FA cells therefore may be the principal cause for BMF and a plausible effect of inefficient clearance of impaired mitochondria. *In-vitro* study of FA cell lines with mitophagy related gene silencing would strengthen the basis of this hypothesis. Together these results shed light on involvement of impaired mitochondria and deregulated mitophagy in FA pathogenesis. This signifies the need of inclusion of mitochondrial nutrients in the management strategies for FA patients.

## Supporting information

S1 Table Primers for gene expression study of OXPHOS complex-I and complex-III encoding and *TFAM* genes. (DOCX)

S2 Table Demographic data, data for chromosomal breakage investigation, FANCD2 immunoblot, and list of *FANCA* (RefSeq#NM_000135) gene mutations. (DOCX)

S3 Table Demographic data, data for chromosomal breakage investigation, FANCD2 immunoblot, and list of *FANCG* gene (RefSeq#NM_004629) mutations. (DOCX)

S4 Table Demographic data, data for chromosomal breakage investigation, FANCD2 immunoblot, and list of *FANCL* gene (RefSeq#NM_018062) mutations. (DOCX)

S5 Table Demographic data, data for chromosomal breakage investigation, FANCD2 immunoblot, and list of *FANCD2* gene (RefSeq# NM_033084) mutations. (DOCX)

S6 Table Demographic data, data for chromosomal breakage investigation, FANCD2 immunoblot, and list of mutations of *FANCB* (RefSeq# NM_001018113), *FANCC* (RefSeq# NM_000136), and *FANCI* (RefSeq# NM_001113378) genes. (DOCX)

S7 Table Demographic data, data for chromosomal breakage investigation and FANCD2 immunoblot for FA patients with mutations not known. (DOCX)

S8 Table Expression fold change of mitophagy genes [*ATG12*, *Beclin1* and *MAP1-LC3*]. (DOCX)

S9 Table Expression fold change of mitophagy genes (*ATG12*, *BECLIN1* and *MAP1-LC3*) in FA patients from different complementation group. (DOCX)

S10 Table OXPHOS complex-I subunits and complex-III encoding gene expression profiling. (DOCX)

S11 Table OXPHOS complex-I subunits and complex-III encoding gene expression profiling in FA patients different complementation groups. (DOCX)

S1 Fig Expression study for *TFAM* gene in FA patients. (A) Fold change for *TFAM* gene expression FA patients, (B) Complementation group-wise comparison of *TFAM* gene expression for FA patients.
(TIF)
