# Supplementary material for: Mitochondrial DNA variations and mitochondrial dysfunction in Fanconi anemia
Source: PLoS One. 2020 Jan 15;15(1):e0227603. doi: 10.1371/journal.pone.0227603 (PMC6961948; doi:10.1371/journal.pone.0227603)
Supplement: S1 Table — (DOCX) [file pone.0227603.s001.docx]

**Supplementary information**

**S1 Table. Primers for gene expression study of OXPHOS complex-I and complex-III encoding and *TFAM* genes.**

| Gene | Primer Sequence |
| --- | --- |
| CYTB_F  CYTB_R | TATCCGCCATCCCATACATT  GGTGATTCCTAGGGGGTTGT |
| ND1_F  ND1_R | CTACTACAACCCTTCGCTGAC  GGATTGAGTAAACGGCTAGGC |
| ND2_F  ND2_R | CATATACCAAATCTCTCCCTC  GTGCGAGATAGTAGTAGGGTC |
| ND3_F  ND3_R | TTACGAGTGCGGCTTCGACC  CCTAGTTTTAAGAGTACTGCG |
| ND4_F  ND4_R | CTAGGCTCACTAAACATTCTA  CCTAGTTTTAAGAGTACTGCG |
| ND4L_F  ND4L_R | TAGTATATCGCTCACACCTC  GTAGTCTAGGCCATATGTG |
| ND5_F  ND5_R | TCGAATAATTCTTCTCACCC  TAGTAATGAGAAATCCTGCG |
| ND6_F  ND6_R | GTAGGATTGGTGCTGTGG  GGATCCTCCCGAATCAAC |
| TFAM_F  TFAM_R | AGCGTTGGAGGGAACTTCCT  CTGACTTGGAGTTAGCTGTTC |
| GAPDH_F  GAPDH_R | GAAGGTGAAGGTCGGAGTC  GAAGATGGTGATGGGATTTC |
| ACTIN_F  ACTIN_R | CGCGAGAAGATGACCCAGAT  TCACCGGAGTCCATCACGAT |
