# Supplementary material for: Mitochondrial DNA variations and mitochondrial dysfunction in Fanconi anemia
Source: PLoS One. 2020 Jan 15;15(1):e0227603. doi: 10.1371/journal.pone.0227603 (PMC6961948; doi:10.1371/journal.pone.0227603)
Supplement: S2 Table — (DOCX) [file pone.0227603.s002.docx]

**Supplementary information**

**S2 Table. Demographic data, data for chromosomal breakage investigation, FANCD2 immunoblot, and list of *FANCA* (RefSeq#NM_000135) gene mutations.**

| **Age** | **Gender** | **Chromosomal breakage score** | **FANCD2 Immunoblotting** | **Exon/**  **Intron** | **Allele 1** | **Protein change** | **Exon/Intron** | **Allele 2** | **Protein change** |
| --- | --- | --- | --- | --- | --- | --- | --- | --- | --- |
| 11 | M | 7.11breaks/metaphase | S-form FANCD2 only | 28 | c.2749C>T | p.R917X | 28 | c.2749C>T | p.R917X |
| 9 | M | 11.24breaks/ metaphase | S-form FANCD2 only | 6 | Exon 6 deletion |  | 6 | Exon 6 deletion |  |
| 6 | F | 2.318breaks/metaphase | S-form FANCD2 only | 32 | c.3239G>A | p.R1080Q | 32 | c.3239G>A | p.R1080Q |
| 8 | M | 7.67breaks/metaphase | S-form FANCD2 only | 20 | c.1795delC | p.S599SfsX5 | 20 | c.1795delC | p.S599SfsX5 |
| 9 | M | 1.893 breaks/ metaphase | S-form FANCD2 only | 29-43 | microdeletion | --- | 41 | c.4085T>A | p.L1362X |
| 14 | M | 1.49breaks/metaphase | S-form FANCD2 only | 39 | c.3926_3929delCAGA | p.T1309Rfs52 | 39 | c.3926_3929delCAGA | p.T1309Rfs52 |
| 11 | M | 10.1breaks/metaphase | S-form FANCD2 only | 33 | c.3282G>C | p.R1084P | IVS 39 | c.3934 +2 T>C | p.S1277TfsX51 |
| 13 | M | 4.81breaks/metaphase | S-form FANCD2 only | 36 | c.3538G>A | p.V1180M | 36 | c.3538G>A | p.V1180M |
| 27 | M | 1.533breaks/metaphase | S-form FANCD2 only | 26 | c.2499delC | p.C833CfsX | 26 | c.2499delC | p.C833CfsX |
| 14 | M | 6breaks/metaphase | S-form FANCD2 only | 28 | c.2656G>T | p.E886X | 28 | c.2656G>T | p.E886X |
| 4 | F | 5.86breaks/metaphase | S-form FANCD2 only | 37 | c.3745delC | p.L1249WfsX9 | 37 | c.3745delC | p.L1249WfsX9 |
| 32 | F | 5.69breaks/metaphase | S-form FANCD2 only | IVS 9 | c.826+2T>C |  | IVS 9 | c.826+2T>C |  |
| 11 | M | 6.95breaks/metaphase | S-form FANCD2 only | 24 | c.2182C>T | p.Q728X | 24 | c.2182C>T | p.Q728X |
| 4.9 | F | 10 breaks/ metaphase | S-form FANCD2 only | 14 | c.1303 C>T | p.R435C | 33 | c.3263C>T | p.S1088F |
| 16 | M | 5.21breaks/metaphase | S-form FANCD2 only | 37 | c.3679G>C | p.A1227P | 37 | c.3679G>C | p.A1227P |
| 5 | M | 4.15breaks/metaphase | S-form FANCD2 only | Exon 12 to 43 | biallelic deletion of FANCA |  | Exon 12 to 43 | biallelic deletion of FANCA |  |
| 15 | F | 6.09breaks/metaphase | S-form FANCD2 only | 27 | c.2559_2564delAGATAC | p.R853RfsX11 | 27 | c.2559_2564delAGATAC | p.R853RfsX11 |
| 5 | F | 6.0 breaks/metaphase | S-form FANCD2 only | IVS 31 | c.3066+1G>T | p.S994RfsX3 | IVS 31 | c.3066+1G>T | p.S994RfsX3 |
| 10 | F | 1.64breaks/metaphase | S-form FANCD2 only | 32 | c.3189G>A | p.W1063X | 32 | c.3189G>A | p.W1063X |
| 10 | F | 2.7breaks/metaphase | S-form FANCD2 only | IVS 39 | c.3934 +2 T>C | p.S1277TfsX51 | IVS 39 | c.3934 +2 T>C | p.S1277TfsX51 |
| 34 | M | 5.69breaks/metaphase | S-form FANCD2 only | 11 | c.987_990delTCAC | p.T329TfsX4 | 11 | c.987_990delTCAC | p.T329TfsX4 |
| 13 | F | 2.4breaks/metaphase | S-form FANCD2 only | 30 | c.2884_2885delCT | p.P965RfsX8 | 30 | c.2884_2885delCT | p.P965RfsX8 |
| 5 | F | 3.71breaks/metaphase | S-form FANCD2 only | 27 | c.2574C>G | p.S858R | 27 | c.2574C>G | p.S858R |
| 8 | F | 6.01breaks/ metaphase | S-form FANCD2 only | 40 | c.3992T>C | p.L1331P | - | not found yet | not found yet |
| 7 | F | Breakage positive* | S-form FANCD2 only | Exon 1 to 43 | biallelic deletion of FANCA |  | Exon 1 to 43 | biallelic deletion of FANCA |  |
| 8 | M | 4.81breaks/metaphase | S-form FANCD2 only | 29 | c.2851C>T | p.R951W | 29 | c.2851C>T | p.R951W |
| 16 | F | 4.39breaks/metaphase | S-form FANCD2 only | Exon 11 | microdeletion | Exon 11 deletion | Exon 11 | microdeletion | Exon 11 deletion |
| 8 | M | 5.714breaks/metaphase | S-form FANCD2 only | exon 4 to 17 | Exon 4–7 deletion |  | exon 4 to 17 | Exon 4–7 deletion |  |
| 11 | M | 3.69breaks/metaphase | S-form FANCD2 only | 28 | c.2733G>A | p.W911X | - | not found yet | not found yet |
| 15 | M | 3.979breaks/metaphase | S-form FANCD2 only | 15-29 | Exon 15–29 deletion |  | 15-29 | Exon 15–29 deletion |  |
| 3 | M | 3.93breaks/metaphase | S-form FANCD2 only | 26 | c.2499delC | p.L834Xfs1 | 26 | c.2499delC | p.L834Xfs1 |
| 5 | F | 8.25breaks/metaphase | S-form FANCD2 only | 24 | c.2182C>T | p.Q728X | 24 | c.2182C>T | p.Q728X |
| 7.6 | M | 6.53 breaks/ metaphase | S-form FANCD2 only | IVS 32 | c.3239+2 T>G | p.E1023DfsX35 | IVS 32 | c.3239+2 T>G | p.E1023DfsX35 |
| 5.6 | F | 7.53breaks/metaphase | S-form FANCD2 only | Exon 1 to 43 | biallelic deletion of FANCA |  | Exon 1 to 43 | biallelic deletion of FANCA |  |
| 17 | M | 4.56breaks/metaphase | S-form FANCD2 only | 29 | c.2786A>C | p.Y929S | 29 | c.2786A>C | p.Y929S |
| 9 | M | 4.2 breaks/ metaphase | S-form FANCD2 only | 37 | c.3677C>G | p.S1226X | 37 | c.3677C>G | p.S1226X |

* We directly dealt with FANCD2 immunoblot investigation for these patients whose samples were referred to us as Chromosomal breakage positive for FA.
