# Supplementary material for: Mitochondrial DNA variations and mitochondrial dysfunction in Fanconi anemia
Source: PLoS One. 2020 Jan 15;15(1):e0227603. doi: 10.1371/journal.pone.0227603 (PMC6961948; doi:10.1371/journal.pone.0227603)
Supplement: S3 Table — (DOCX) [file pone.0227603.s003.docx]

**Supplementary information**

**S3 Table. Demographic data, data for chromosomal breakage investigation, FANCD2 immunoblot, and list of *FANCG* gene (RefSeq#**[**NM_004629**](http://www.ncbi.nlm.nih.gov/nuccore/NM_004629)**) mutations.**

| **Age** | **Gender** | **Chromosomal breakage score** | **FANCD2 Immunoblotting** | **Exon/**  **Intron** | **Allele 1** | **Protein change** | **Exon/**  **Intron** | **Allele 2** | **Protein change** |
| --- | --- | --- | --- | --- | --- | --- | --- | --- | --- |
| 7 | M | 3.03breaks/metaphase | S-form FANCD2 only | IVS12 | c.1636+7A>G | SKIPPING OF EXON 12 | IVS12 | c.1636+7A>G | SKIPPING OF EXON 12 |
| 5.5 | M | 8.14breaks/metaphase | S-form FANCD2 only | EXON 12 | c.1501C>T | p.Q501X | IVS12 | c.1636+7A>G | SKIPPING OF EXON 12 |
| 5 | F | 6.6 breaks/ metaphase | S-form FANCD2 only | EXON 7 | c.883dupG | p. (Asp295GlyfsX14) | EXON 7 | c.883dupG | p. (Asp295GlyfsX14) |
| 11 | M | 2breaks/metaphase | S-form FANCD2 only | EXON 10 | c.1252G>T | p.E418X | EXON 10 | c.1252G>T | p.E418X |
| 11 | M | 3.57breaks/metaphase | S-form FANCD2 only | EXON 1 | c.77G>A | P.Q26R | IVS12 | c.1636+7A>G | SKIPPING OF EXON 12 |
| 13 | M | 2.21breaks/metaphase | S-form FANCD2 only | EXON 11 | c.1471_1473delAAAinsG | p. (Lys491GlyfsX9) | EXON 11 | c.1471_1473delAAAinsG | p. (Lys491GlyfsX9) |
| NA^#^ | M | 4.97breaks/metaphase | S-form FANCD2 only | EXON5 | c.637_643delTACCGCC | p.Y213KfsX7 | EXON5 | c.637_643delTACCGCC | p.Y213KfsX7 |
| 12 | F | 4.91breaks/metaphase | S-form FANCD2 only | EXON7 | c.787C>T | p.Q263X | EXON7 | c.787C>T | p.Q263X |
| NA^#^ | M | 4.15breaks/metaphase | S-form FANCD2 only | Exon 12 | c.1501C>T | p.Q501X | IVS12 | c.1636+7A>G | p.Q501X |
| 7 | M | Breakage positive* | S-form FANCD2 only | IVS9 | c.1143 + 5G > C | p. (Arg359SerfsX22) | IVS9 | c.1143 + 5G > C | p. (Arg359SerfsX22) |
| 7 | F | 3.92 breaks/ metaphase | S-form FANCD2 only | EXON 10 | c.1375C>T | p.Q459X | EXON 10 | c.1375C>T | p.Q459X |
| 6 | M | 2.96breaks/metaphase | S-form FANCD2 only | EXON 11 | c.1467G>T | p.E490X | EXON 11 | c.1467G>T | p.E490X |

*We directly dealt with FANCD2 immunoblot investigation for these patients whose samples were referred to us as Chromosomal breakage positive for FA.

^#^ Age of this patient was not available.
