# Supplementary material for: Mitochondrial DNA variations and mitochondrial dysfunction in Fanconi anemia
Source: PLoS One. 2020 Jan 15;15(1):e0227603. doi: 10.1371/journal.pone.0227603 (PMC6961948; doi:10.1371/journal.pone.0227603)
Supplement: S4 Table — (DOCX) [file pone.0227603.s004.docx]

**Supplementary information**

**S4 Table. Demographic data, data for chromosomal breakage investigation, FANCD2 immunoblot, and list of *FANCL* gene (RefSeq#NM_018062) mutations.**

| **Age** | **Gender** | **Chromosomal breakage score** | **FANCD2 immunoblot** | **Exon/**  **Intron** | **Allele 1** | **Protein change** | **Exon/**  **Intron** | **Allele 2** | **Protein change** |
| --- | --- | --- | --- | --- | --- | --- | --- | --- | --- |
| 3 | M | 2.6 breaks/ metaphase | S-form FANCD2 only | EXON 13 | c.1092G>A | p.K364K | EXON 13 | c.1092G>A | p.K364K |
| 6 | F | 1.56 breaks/metaphase | S-form FANCD2 only | EXON 13 | c.1092G>A | p.K364K | EXON 8 | c.592delA | p.K198RfsX33 |
| 10 | M | 1.66 breaks/metaphase | S-form FANCD2 only | EXON 13 | c.1092G>A | p.K364K | EXON 13 | c.1092G>A | p.K364K |
| 4 | F | 1.44breaks/metaphase | S-form FANCD2 only | EXON 13 | c.1092G>A | p.K364K | EXON 13 | c.1092G>A | p.K364K |
| 10 | F | 2.3breaks/metaphase | S-form FANCD2 only | EXON 13 | c.1092G>A | p.K364K | EXON 13 | c.1092G>A | p.K364K |
| 28 | M | 1.55breaks/metaphase | S-form FANCD2 only | Exon 2 | c.112C>T | p.Leu38Phe | nf | nf | nf |
| 2.8 | M | Breakage positive* | S-form FANCD2 only | EXON 13 | c.1092G>A | p.K364K | EXON 13 | c.1092G>A | p.K364K |
| 5 | F | 1.18breaks/metaphase | S-form FANCD2 only | EXON 13 | c.1092G>A | p.K364K | EXON 13 | c.1092G>A | p.K364K |
