# Supplementary material for: Mitochondrial DNA variations and mitochondrial dysfunction in Fanconi anemia
Source: PLoS One. 2020 Jan 15;15(1):e0227603. doi: 10.1371/journal.pone.0227603 (PMC6961948; doi:10.1371/journal.pone.0227603)
Supplement: S5 Table — (DOCX) [file pone.0227603.s005.docx]

**Supplementary information**

**S5 Table. Demographic data, data for chromosomal breakage investigation, FANCD2 immunoblot, and list of *FANCD2* gene (RefSeq#** [**NM_033084**](http://www.ncbi.nlm.nih.gov/nuccore/NM_033084)**) mutations.**

| **Age** | **Gender** | **Chromosomal breakage score** | **FANCD2 Immunoblotting** | **Gene** | **Exon/**  **Intron** | **Allele 1** | **Protein change** | **Exon/**  **Intron** | **Allele 2** | **Protein change** |
| --- | --- | --- | --- | --- | --- | --- | --- | --- | --- | --- |
| 5 | F | 4.06 breaks/ metaphase | S-form and L-form of FANCD2 | FANCD2 | exon 38 | c.3817C>T | p.R1273X | IVS30 | c.2976+5G>A | In-frame deletion of 39 amino acid |
| 7 | M | 0.85breaks/metaphase | S-form and L-form of FANCD2 | FANCD2 | IVS 30 | c.2976+5G>A | In-frame deletion (Deletion of 39 amino acid) | IVS30 | c.2976+5G>A | In-frame deletion (Deletion of 39 amino acid) |
| 2 | F | 4.625breaks/metaphase | S-form and L-form of FANCD2 | FANCD2 | exon 7 | c.473C>G | p.P158R | Exon 7 | c.473C>G | p.P158R |
