# Supplementary material for: Mitochondrial DNA variations and mitochondrial dysfunction in Fanconi anemia
Source: PLoS One. 2020 Jan 15;15(1):e0227603. doi: 10.1371/journal.pone.0227603 (PMC6961948; doi:10.1371/journal.pone.0227603)
Supplement: S6 Table — (DOCX) [file pone.0227603.s006.docx]

**Supplementary information**

**S6 Table. Demographic data, data for chromosomal breakage investigation, FANCD2 immunoblot, and list of mutations of *FANCB* (RefSeq# NM_001018113), *FANCC* (RefSeq# NM_000136), and *FANCI* (RefSeq# NM_001113378) genes.**

| **Age** | **Gender** | **Chromosomal breakage score** | **FANCD2 Immunoblotting** | **Gene** | **Exon/**  **Intron** | **Allele 1** | **Protein change** | **Exon/**  **Intron** | **Allele 2** | **Protein change** |
| --- | --- | --- | --- | --- | --- | --- | --- | --- | --- | --- |
| 8 | M | 4.0breaks/metaphase | S-form FANCD2 only | FANCI | Exon 18 | c.1813C>T | p.L650F | - | - | - |
| 4 | M | 2.11breaks/metaphase | S-form FANCD2 only | FANCB | Exon 9 | c.2165+2T>G | p.E643Gfs18X | Exon 9 | c.2165+2T>G | p.E643Gfs18X |
| 3 | M | 5.04 breaks/ metaphase | S-form FANCD2 only | FANCC | Exon 15 | c.1642C>T | p.R548X | Exon 15 | c.1642C>T | p.R548X |
