# Supplementary material for: Mitochondrial DNA variations and mitochondrial dysfunction in Fanconi anemia
Source: PLoS One. 2020 Jan 15;15(1):e0227603. doi: 10.1371/journal.pone.0227603 (PMC6961948; doi:10.1371/journal.pone.0227603)
Supplement: S7 Table — (DOCX) [file pone.0227603.s007.docx]

**Supplementary information**

**S7 Table. Demographic data, data for chromosomal breakage investigation and FANCD2 immunoblot for FA patients with mutations not known**

| **Age** | **Gender** | **Chromosomal breakage score** | **FANCD2 Immunoblotting** |
| --- | --- | --- | --- |
| 12 | F | 9.48 breaks/ metaphase | S-form FANCD2 only |
| 11 | M | 4.54breaks/metaphase | S-form FANCD2 only |
| 8 | F | 13.92breaks/metaphase | S-form FANCD2 only |
| 7 | F | 7.83breaks/metaphase | S-form FANCD2 only |
| 5.5 | M | 1.89breaks/metaphase | S-form and L-form of FANCD2 |
| 15 | F | 4.69breaks/metaphase | S-form FANCD2 only |
| 14 | M | 2.23breaks/metaphase | S-form and L-form of FANCD2 |
| NA^#^ | M | 3.44breaks/metaphase | S-form FANCD2 only |

^#^ Age of this patient was not available.
