# Supplementary material for: Mitochondrial DNA variations and mitochondrial dysfunction in Fanconi anemia
Source: PLoS One. 2020 Jan 15;15(1):e0227603. doi: 10.1371/journal.pone.0227603 (PMC6961948; doi:10.1371/journal.pone.0227603)
Supplement: S8 Table — (DOCX) [file pone.0227603.s008.docx]

**Supplementary information**

**S8 Table. Expression fold change of mitophagy genes [*ATG12*, *Beclin1* and *MAP1-LC3*].**

|  | *ATG12* | *Beclin1* | *MAP1-LC3* |
| --- | --- | --- | --- |
| Fold change | 1.049045 | 0.981038 | 2.915251 |
| p-values | >0.05 | >0.05 | 0.02019 |
