# Supplementary material for: Mitochondrial DNA variations and mitochondrial dysfunction in Fanconi anemia
Source: PLoS One. 2020 Jan 15;15(1):e0227603. doi: 10.1371/journal.pone.0227603 (PMC6961948; doi:10.1371/journal.pone.0227603)
Supplement: S9 Table — (DOCX) [file pone.0227603.s009.docx]

**Supplementary information**

**S9 Table. Expression fold change of mitophagy genes (*ATG12*, *BECLIN1* and *MAP1-LC3*) in FA patients from different complementation group**.

|  | *ATG12* | *BECLIN1* | *MAP1-LC3* | p-values | | |
| --- | --- | --- | --- | --- | --- | --- |
|  |  |  |  | ATG12 | *BECLIN1* | *MAP1-LC3* |
| FAA | 1.119264 | 1.146776 | 2.595129 | 0.287091 | 0.31326 | 0.08234 |
| FAG | 1.293221 | 1.342385 | 3.345928 | 0.18049 | 0.212196 | 0.07426 |
| FAL | 1.228644 | 1.384813 | 3.60345 | 0.2023 | 0.173344 | 0.02019 |
