# Supplementary material for: Mitochondrial DNA variations and mitochondrial dysfunction in Fanconi anemia
Source: PLoS One. 2020 Jan 15;15(1):e0227603. doi: 10.1371/journal.pone.0227603 (PMC6961948; doi:10.1371/journal.pone.0227603)
Supplement: S10 Table — (DOCX) [file pone.0227603.s010.docx]

**Supplementary information**

| Genes | Log(RQ) | t-value | p-value |
| --- | --- | --- | --- |
| *ND1* | -0.42201 | 2.50395 | 0.0132 |
| *ND2* | -0.4903 | 2.82347 | 0.0072 |
| *ND3* | -0.79886 | 2.21358 | 0.02268 |
| *ND4* | -0.44289 | 2.34903 | 0.01765 |
| *ND4L* | -0.45785 | 2.69758 | 0.00914 |
| *ND5* | -0.39567 | 1.82982 | 0.04515 |
| *ND6* | -0.40921 | 2.19754 | 0.02335 |
| *CYTB* | -0.4173 | 2.42241 | 0.01538 |

**S10 Table. OXPHOS complex-I subunits and complex-III encoding gene expression profiling.**
