# Supplementary material for: Mitochondrial DNA variations and mitochondrial dysfunction in Fanconi anemia
Source: PLoS One. 2020 Jan 15;15(1):e0227603. doi: 10.1371/journal.pone.0227603 (PMC6961948; doi:10.1371/journal.pone.0227603)
Supplement: S11 Table — (DOCX) [file pone.0227603.s011.docx]

**Supplementary information**

**S11 Table. OXPHOS complex-I subunits and complex-III encoding gene expression profiling in FA patients different complementation groups.**

| Log(RQ) | FAL | FAA | FAG | p-value | | |
| --- | --- | --- | --- | --- | --- | --- |
|  |  |  |  | FAL | FAA | FAG |
| *ND1* | -0.266 | -0.054 | -0.695 | 0.05 | 0.14 | 0.06 |
| *ND2* | -0.400 | 0.060 | -0.688 | 0.00 | 0.15 | 0.06 |
| *ND3* | -0.376 | -0.547 | -0.996 | 0.07 | 0.05 | 0.23 |
| *ND4* | -0.169 | 0.055 | -0.640 | 0.04 | 0.06 | 0.06 |
| *ND4L* | -0.342 | -0.011 | -0.516 | 0.02 | 0.46 | 0.106 |
| *ND5* | -0.106 | 0.102 | -0.653 | 0.13 | 0.00 | 0.141 |
| *ND6* | -0.394 | 0.021 | -0.744 | 0.00 | 0.31 | 0.12 |
| *CYTB* | -0.246 | 0.083 | -0.644 | 0.046 | 0.20 | 0.06 |
