# Supplementary figures and images for: Mitochondrial DNA variations and mitochondrial dysfunction in Fanconi anemia
Source: PLoS One. 2020 Jan 15;15(1):e0227603. doi: 10.1371/journal.pone.0227603 (PMC6961948; doi:10.1371/journal.pone.0227603)

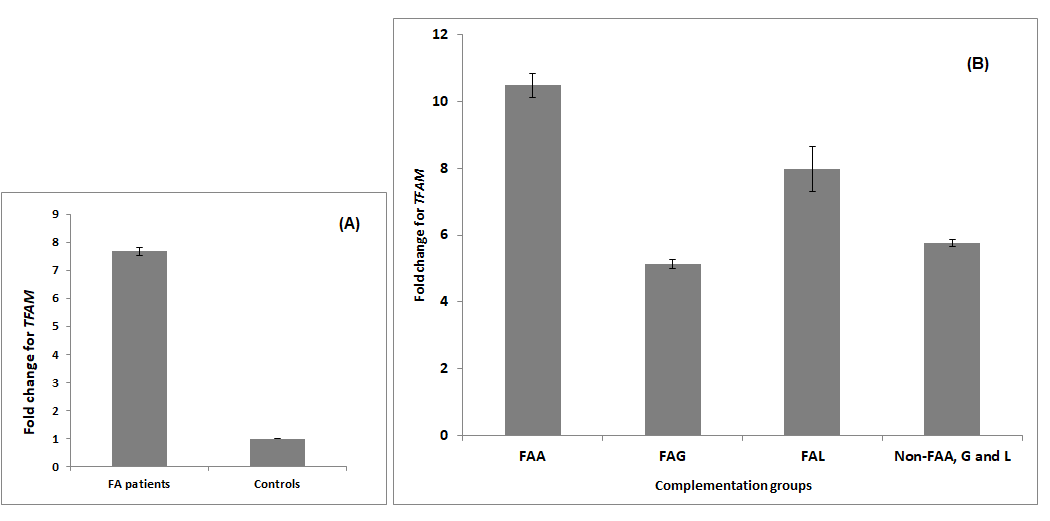

Supplement: S1 Fig — (A) Fold change for TFAM gene expression FA patients, (B) Complementation group-wise comparison of TFAM gene expression for FA patients. (TIF) [file pone.0227603.s012.tif]
